# Research on Omnidirectional Gait Switching and Attitude Control in Hexapod Robots

**DOI:** 10.3390/biomimetics9120729

**Published:** 2024-11-29

**Authors:** Min Yue, Xiaoyun Jiang, Liqiang Zhang, Yujin Zhang

**Affiliations:** 1 School of Mechanical and Automotive Engineering, Shanghai University of Engineering Science, Shanghai 201620, China; sui_bkt@outlook.com (X.J.); zhanglq@sues.edu.cn (L.Z.); 2 School of Electronic and Electrical Engineering, Shanghai University of Engineering Science, Shanghai 201620, China; yjzhang@sues.edu.cn

**Keywords:** hexapod robot, gait switching, attitude control, fuzzy inference, single-neuron adaptive PID

## Abstract

To tackle the challenges of poor stability during real-time random gait switching and precise trajectory control for hexapod robots under limited stride and steering conditions, a novel real-time replanning gait switching control strategy based on an omnidirectional gait and fuzzy inference is proposed, along with an attitude control method based on the single-neuron adaptive proportional–integral–derivative (PID). To start, a kinematic model of a hexapod robot was developed through the Denavit–Hartenberg (D-H) kinematics analysis, linking joint movement parameters to the end foot’s endpoint pose, which formed the foundation for designing various gaits, including omnidirectional and compound gaits. Incorporating an omnidirectional gait could effectively resolve the challenge of precise trajectory control for the hexapod robot under limited stride and steering conditions. Next, a real-time replanning gait switching strategy based on an omnidirectional gait and fuzzy inference was introduced to tackle the issue of significant impacts and low stability encountered during gait transitions. Finally, in view of further enhancing the stability of the hexapod robot, an attitude adjustment algorithm based on the single-neuron adaptive PID was presented. Extensive experiments confirmed the effectiveness of this approach. The results show that our approach enabled the robot to switch gaits seamlessly in real time, effectively addressing the challenge of precise trajectory control under limited stride and steering conditions; moreover, it significantly improved the hexapod robot’s dynamic stability during its motion, enabling it to adapt to complex and changing environments.

## 1. Introduction

The demand for autonomous mobile robots is increasingly growing with the rapid advancement of technology and the accelerated pace of human exploration and development of the natural world [1,2]. The primary categories of autonomous mobile robots are wheeled, tracked, and legged robots, each engineered to perform optimally in specific environments and scenarios [3]. Wheeled mobile robots are equipped with wheels at their base, allowing for fast movement and high efficiency [4]. However, they are limited to flat surfaces, and their adaptability and obstacle-crossing capabilities are relatively poor [5]. Tracked mobile robots feature track assemblies at their base, enhancing their load capacity and durability to adapt to multiple types of terrain [6]. Nevertheless, these tracks are cumbersome, leading to higher maintenance expenses and slower speeds. Legged mobile robots are designed to mimic the gait of humans, dogs, and spiders to progress through complex stepping motions, categorized into biped, three-legged, quadruped, hexapod, and multi-legged robots [7,8,9,10,11]. Compared to biped, three-legged, and quadruped robots, hexapod robots offer significant advantages in load capacity and dynamic stability with their redundant leg structures and multiple contact points to distribute weight [12]. This makes them particularly suitable for autonomous navigation over uneven and complex terrain. Consequently, they are extensively utilized in diverse applications such as disaster response, military reconnaissance and combat, and resource exploration [13,14,15].

Hexapod robots’ numerous joints and legs provide them with a high degree of redundancy, which presents significant challenges for motion control [16]. In general, effective gait switching and attitude control algorithms are viewed as essential for hexapod robots to effectively handle complex tasks in challenging environments [17,18]. Mao et al. [19] proposed a gait switching approach that flexibly generates multiple gaits for continuous non-differentiable terrain. This method has already been implemented in the Hexa-XIII hexapod robot, enabling it to climb stairs with inclines exceeding 45°. Bal [20] presented a smooth gait transition algorithm based on a central pattern generator (CPG) and locomotion control strategy, which can address suboptimal leg movements during the process of gait transition. Luneckas et al. [21] introduced a heuristic algorithm for gait switching based on a robot’s current speed, aimed at minimizing energy consumption. Chen et al. [22] developed a hierarchical control framework for gait switching, employing a flexible gait planner and gait feedback regulator. This method enables a robot to select the optimal foothold for adapting to unstructured terrain. Chen et al. [23] detailed a novel gait switching algorithm that enables a robot to seamlessly switch between gaits during movement, improving its capability to handle challenging Martian terrain. Zhang et al. [24] introduced a novel CPG–fuzzy heading control approach for a blade-legged hexapod robot. This method innovatively combines CPG models with fuzzy control to achieve real-time heading adjustments. However, this method only applies to the 6-DOF blade-legged robot and does not address the issue of real-time gait transitions. Currently, most of these studies predominantly focus on switching between single gaits. Furthermore, these transitions generally commence only after the current gait cycle has been completed, ensuring a robot’s stability. However, there is a noticeable lack of emphasis on the real-time switching among multiple gaits in hexapod robots, which is crucial for dynamic adaptability in complex environments.

The attitude adjustment strategy for the hexapod robot is significant, as it enhances stability and improves the robot’s adaptability to various types of unstructured terrain, thereby increasing its overall robustness. Many scholars have made efforts in this regard. Chen et al. [25] introduced an attitude trajectory optimization algorithm that employs higher-order polynomial interpolation to reduce attitude fluctuations during a robot’s operation, facilitating effective attitude adjustment. However, this method involves several parameters that require user tuning, which must be set according to the desired performance in specific scenarios. This makes the tuning process difficult, and improper adjustments can lead to undesirable issues, such as a robot shaking. Coelho et al. [26] adopted limb positions to predict the ground slope and computed novel limb trajectories to adjust the attitude angles of a hexapod robot. However, this approach has only been tested through computer simulations and has not yet been validated in real-world scenarios.

Although gait switching and attitude control enhance the stability and smoothness of a hexapod robot’s movement, achieving precise trajectory control and more complex maneuvers on unstructured terrain remains challenging. However, omnidirectional movement enables a robot to move in any direction without altering its heading direction, making it an ideal solution. Tang et al. [27] introduced an omnidirectional gait for a passive vertebrate hexapod robot consisting of three body segments connected by passive joints. This approach enables an omnidirectional gait by adjusting the duration of each switching configuration. Wang et al. [28] achieved omnidirectional motion based on CPG bottom feedback and studied transitional movement from flat to sloped land. In a similar approach, Wang et al. [29] presented an omnidirectional gait based on a CPG and dynamic thresholds for a hexapod robot to navigate through non-structured environments. However, the omnidirectional gait mentioned above does not provide a strategy for real-time switching between multiple gaits.

In this article, a novel real-time replanning gait switching control strategy based on omnidirectional gait and fuzzy inference is proposed, along with an attitude control method based on the single-neuron adaptive PID algorithm. This hexapod robot leverages a fuzzy inference model, incorporating orientation deviation and velocity deviation factors, to facilitate the real-time calculation and switching of gaits while ensuring stability. Furthermore, it utilizes a single-neuron adaptive PID attitude control algorithm to control its attitude autonomously. The primary contributions of this paper can be summarized as follows:

1. Several gait patterns are developed by utilizing the tripod gait based on kinematic analysis, including forward and omnidirectional, rotational, and compound patterns. Incorporating an omnidirectional gait can effectively resolve the challenge of precise trajectory control for the hexapod robot under limited stride and steering conditions.
2. A novel method for omnidirectional gait switching based on a fuzzy inference algorithm is proposed. This approach enables the hexapod robot to seamlessly switch to the most appropriate gait in real time without the need to complete the current gait, enhancing the stability of gait switching and, thus, improving operational efficiency across diverse environments.
3. An autonomous attitude control method is introduced based on the single-neuron adaptive PID control algorithm. This approach enables the robot to autonomously and dynamically adjust its controller parameters online, enhancing its dynamic stability during its motion and thereby adapting to complex and variable environments.

The remainder of this paper is organized as follows: Section 2 introduces the structural design of the hexapod robot. Section 3 details the kinematic analysis performed on the hexapod robot using the D-H method. The subsequent section outlines each gait pattern based on the tripod gait for the hexapod robot, along with autonomous gait switching based on a fuzzy inference algorithm. Section 5 introduces an autonomous attitude adjustment algorithm based on a single-neuron adaptive PID. Four sets of experiments were conducted to verify the effectiveness of our proposed approaches, as presented in Section 6. The final section summarizes the principal findings of this paper and provides an outlook on future work.

## 2. Structure Design

**Figure 1:**
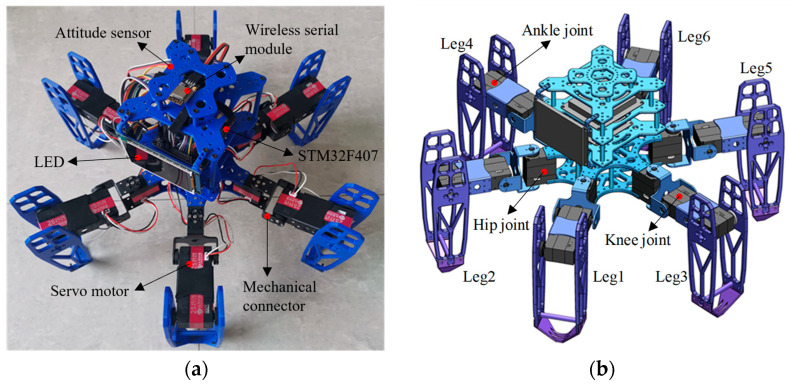
The hexapod robot: (**a**) the prototype; (**b**) the simulation model diagram of the hexapod robot.

The distribution patterns of legs in hexapod or multi-legged organisms primarily include radial symmetry and uniform distribution. During walking, the center-of-mass projection consistently falls within the area formed by the lines connecting the supporting legs. Consequently, the leg distribution of our designed hexapod robot employs a uniform symmetrical arrangement. We define the head as the forward direction. For reference, the two legs closest to the head are labeled as Leg1 and Leg2. The remaining legs are numbered in an axisymmetric interval order. The prototype and simulation model diagram of the hexapod robot are illustrated in Figure 1.

The mechanical structure of the hexapod robot is primarily composed of its main body and six legs, where the leg structure is complex. To ensure stable movement while simplifying the leg structure, each leg consists of a coxa (base joint), femur (thigh), and tibia (shin). These legs are equipped with rotatable hip, knee, and ankle joints, all driven by digital servos. Therefore, each leg features three degrees of freedom: a base joint capable of horizontal rotation and two joints in the thigh and shin allowing for vertical rotation. Additionally, the leg modules employ a modular, hollow skeletal design, achieving excellent interchangeability and lightweight construction. These design choices make the legs more compact and reduce potential conflicts between connecting wires. The main parameters of the hexapod robot are illustrated in Table 1.

## 3. Kinematic Analysis

**Figure 2:**
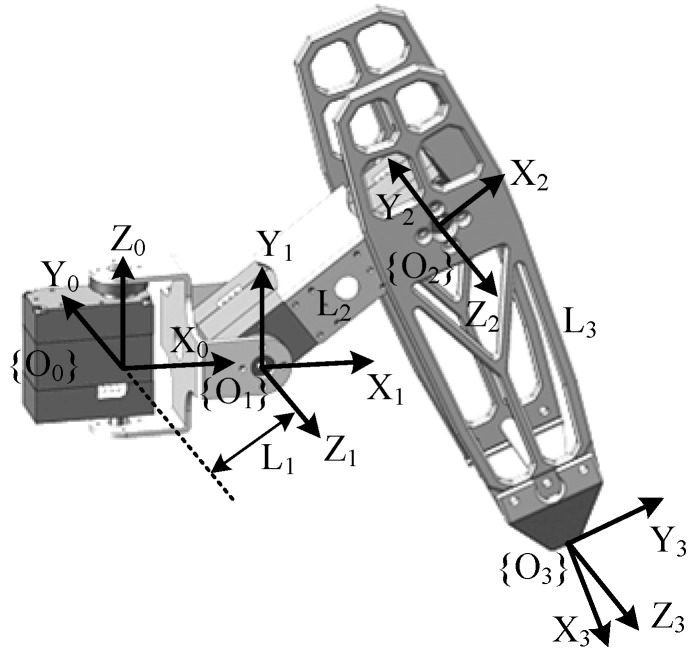
The coordinate frames of the robot’s leg, with 3 degrees of freedom.

Each leg of the hexapod robot can be modeled as a three-link serial mechanism. To mimic the movement patterns of insects and achieve diverse gaits and attitude control, it is necessary to perform kinematic analysis and coordinate the movements of all of the joints across the six legs. The establishment and solution of the robot’s forward and inverse kinematics are fundamental prerequisites for effective motion control. According to the classic D-H method [30,31], the coordinate system for each joint can be established, as illustrated in Figure 2. Additionally, the parameters of the D-H method are shown in Table 2.

### 3.1. The Forward Kinematics

The forward kinematics of each leg are determined by calculating the coordinate of the foot’s endpoint in the fixed coordinate system {O_0_} of the base joint, based on the known angles of the drive joints. This process fundamentally involves the translation and rotation transformations of the link coordinate systems. According to the D-H parameter method, the transformation matrix $T_{i-1}^{i}$ for each link can be expressed as follows [15]:

(1) $$T_{i-1}^{i}=\left[\begin{array}{cccc} \cos\left(\theta_{i}\right) & -\sin\left(\theta_{i}\right)\cos\left(\alpha_{i}\right) & \sin\left(\theta_{i}\right)\sin\left(\alpha_{i}\right) & a_{i}\cos\left(\theta_{i}\right)\\ \sin\left(\theta_{i}\right) & \cos\left(\theta_{i}\right)\cos\left(\alpha_{i}\right) & -\cos\left(\theta_{i}\right)\sin\left(\alpha_{i}\right) & a_{i}\sin\left(\theta_{i}\right)\\ 0 & \sin\left(\alpha_{i}\right) & \cos\left(\alpha_{i}\right) & d_{i}\\ 0 & 0 & 0 & 1 \end{array}\right]$$

According to Table 2, the corresponding D-H parameters for each link can be substituted into Equation (1) to derive the transformation matrix $T_{i-1}^{i}$ for each link. The continuous transformation formula for the links is as follows:

(2) $$T_{0}^{3}=T_{0}^{1}T_{1}^{2}T_{2}^{3}$$

By applying Equation (2), the transformation matrix expression for the endpoint coordinate system {O_3_} relative to the base joint coordinate system {O_0_} can be derived.

(3) $$T_{0}^{3}=\left[\begin{array}{cccc} \cos\left(\theta_{1}\right)\cos\left(\theta_{2}+\theta_{3}\right) & -\cos\left(\theta_{1}\right)\sin\left(\theta_{2}+\theta_{3}\right) & \sin\left(\theta_{1}\right) & \left(L_{1}+L_{2}\cos\left(\theta_{2}\right)+L_{3}\cos\left(\theta_{3}+\theta_{2}\right)\right)\cos(\theta_{1})\\ \sin\left(\theta_{1}\right)\cos\left(\theta_{2}+\theta_{3}\right) & -\sin\left(\theta_{1}\right)\sin\left(\theta_{2}+\theta_{3}\right) & -\cos\left(\theta_{1}\right) & \left(L_{1}+L_{2}\cos\left(\theta_{2}\right)+L_{3}\cos\left(\theta_{3}+\theta_{2}\right)\right)\sin(\theta_{1})\\ \sin\left(\theta_{2}+\theta_{3}\right) & \cos\left(\theta_{2}+\theta_{3}\right) & 0 & L_{2}\sin\left(\theta_{2}\right)+L_{3}\sin\left(\theta_{3}+\theta_{2}\right)\\ 0 & 0 & 0 & 1 \end{array}\right]$$

The fourth column vector represents the offset of the leg’s endpoint within the base joint coordinate system. Thus, the forward kinematics expression for a single leg is as follows:

(4) $$\left[\begin{array}{c} x\\ y\\ z \end{array}\right]=\left[\begin{array}{c} \left(L_{1}+L_{2}\cos\left(\theta_{2}\right)+L_{3}\cos\left(\theta_{3}+\theta_{2}\right)\right)\cos(\theta_{1})\\ \left(L_{1}+L_{2}\cos\left(\theta_{2}\right)+L_{3}\cos\left(\theta_{3}+\theta_{2}\right)\right)\sin(\theta_{1})\\ L_{2}\sin\left(\theta_{2}\right)+L_{3}\sin\left(\theta_{3}+\theta_{2}\right) \end{array}\right]$$

where (*x*, *y*, *z*) refers to the coordinates of the foot’s endpoint relative to the coordinate system of the base joint.

### 3.2. The Inverse Kinematics

The calculations for inverse kinematics are based on forward kinematics. By utilizing inverse kinematics, the necessary joint angles for each leg can be determined based on the known coordinates of the foot endpoints. Inverse kinematics is fundamental for controlling leg movements in hexapod robots, ensuring smooth and accurate leg movements. We assume that the coordinates of the foot’s endpoint p are as follows:

(5) $$p=\left[\begin{array}{c} x\\ y\\ z \end{array}\right]$$

According to Equation (4), the required joint angles for each leg can be calculated as follows:

(6) $$\left[\begin{array}{c} \theta_{1}\\ \theta_{2}\\ \theta_{3} \end{array}\right]=\left[\begin{array}{c} \mathrm{atan}2\left(y,x\right)\\ -\mathrm{atan}2\left(-z,L-L_{1}\right)+\arccos\left(\frac{z^{2}+{\left(L-L_{1}\right)}^{2}+{L_{2}}^{2}-{L_{3}}^{2}}{2L_{2}\sqrt{z^{2}+{\left(L-L_{1}\right)}^{2}}}\right)\\ -\arccos\left(\frac{z^{2}+{\left(L-L_{1}\right)}^{2}-{L_{2}}^{2}+{L_{3}}^{2}}{2L_{3}\sqrt{z^{2}+{\left(L-L_{1}\right)}^{2}}}\right)-\arccos\left(\frac{z^{2}+{\left(L-L_{1}\right)}^{2}+{L_{2}}^{2}-{L_{3}}^{2}}{2L_{2}\sqrt{z^{2}+{\left(L-L_{1}\right)}^{2}}}\right) \end{array}\right]$$

(7) $$L=\sqrt{x^{2}+y^{2}}$$

## 4. Gait Planning and Gait Switching Strategy

Hexapod robots utilize a variety of insect-inspired gaits, primarily including tripod, quadrangular, and pentagonal gaits [32]. In the tripod gait, three legs can swing simultaneously, whereas in the quadrangular and pentagonal gaits, only two legs and one leg can swing at a time, respectively. Therefore, in the tripod gait, the load capacity of the hexapod robot is slightly lower than that under the other two gaits, but it achieves higher speed and greater walking efficiency. All gaits discussed in this paper are based on the tripod gait.

**Figure 3:**
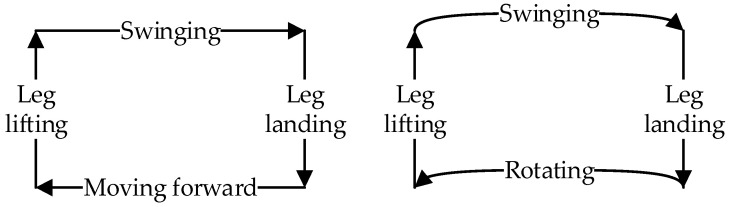
The gait cycle of the hexapod robot.

When the hexapod robot operates in this gait, its six legs are divided into two groups. As illustrated in Figure 1, for the convenience of describing the operation of each gait, Leg1 and Leg5 on the left and Leg4 on the right are recorded as group G1, while Leg2 and Leg6 on the right and Leg3 on the left are recorded as group G2. By dividing the six legs into two groups, they create two centrosymmetric triangular support structures, ensuring that the robot’s center of gravity always remains within the triangle formed by the support group’s feet, thereby supporting the robot’s entire body. The legs in direct contact with the ground are termed the support group, while those not in contact with the ground are referred to as the swing group. When the hexapod robot moves, its gait follows a cyclical pattern. Each movement can be decomposed into four distinct states, forming a complete cycle together. By linking multiple cycles, the robot can achieve a variety of complex gaits, enabling it to adapt to different terrain and tasks. The gait cycle of a hexapod robot can be broadly categorized into four states: leg lifting, swinging, leg landing, and moving, as illustrated in Figure 3.

1. Leg-lifting state: The initial support group elevates, transitioning into the swing group.
2. Swinging state: The swing group moves from the starting position to the endpoint.
3. Leg-landing state: The initial swing group descends, contacting the ground to become the new support group.
4. Moving state: The support group moves from the starting position to the endpoint. This movement propels the entire robot forward, as the foot-ends of the support group remain stationary relative to the ground. Depending on the movement mode, this state can be further categorized into a forward and omnidirectional gait, rotational gait, and compound gait.

### 4.1. Forward and Omnidirectional Gait

**Figure 4:**
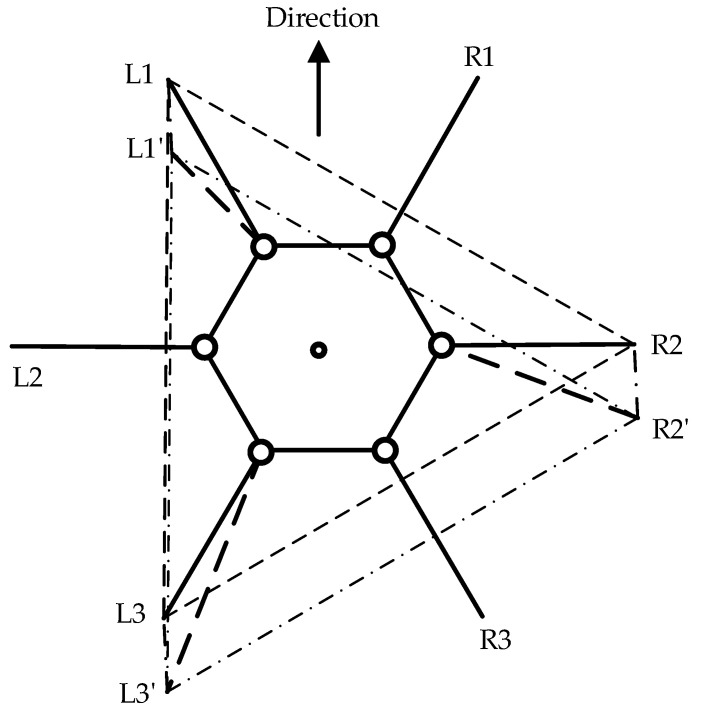
Displacement diagram of the support triangle in the forward gait, where L1, L2, and L3 denote the endpoints of Leg1, Leg3, and Leg5, respectively, while R1, R2, and R3 represent the endpoints of Leg2, Leg4, and Leg6, respectively. The support triangle for the hexapod robot before forward motion is formed by L1, R2, and L3, while L1′, R2′, and L3′ represent the support triangle after the robot undergoes relative movement.

**Figure 5:**
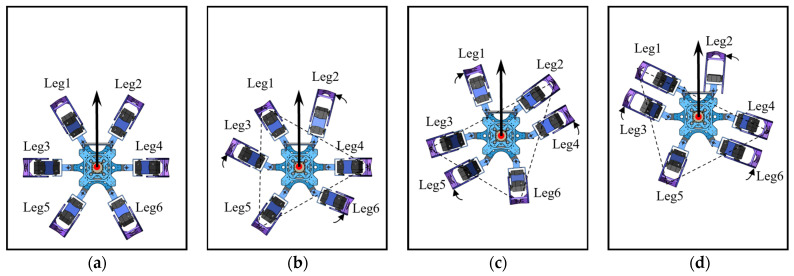
The forward gait of the hexapod robot: (**a**) the robot’s initial state; (**b**) the robot’s status with G1 as the support group; (**c**) the robot’s status with G2 as the support group; (**d**) the robot’s status with G1 as the support group.

The forward gait and omnidirectional gait are both referred to as parallel displacement gaits. The forward gait is the robot’s fundamental motion pattern, which is relatively simple to implement without considering most constraints. However, to maintain stability during movement, trajectory planning is necessary to determine the leg movements. As illustrated in Figure 4, the support triangle formed by the feet of the support group was analyzed, revealing that the support triangle remains stationary relative to the ground during the linear movement of the hexapod robot. Additionally, the support triangle moves in a straight line relative to the robot, opposite to the robot’s direction of motion. Consequently, in the robot’s forward gait, the movement trajectories of the support group’s feet are parallel to the robot’s movement trajectory, with a direction opposite to the robot’s movement and a trajectory length equal to the robot’s stride length. Therefore, in the forward gait, it is essential to maintain L1L1′, L3L3′, and R2R2′ that are parallel and of equal length. Consequently, the robot’s movement direction relative to the ground aligns with the vector L1′L1, and the forward distance equals the length of L1L1′. The forward motion of the hexapod robot is achieved through the periodic interchange of the support and swing groups, as illustrated in Figure 5.

**Figure 6:**
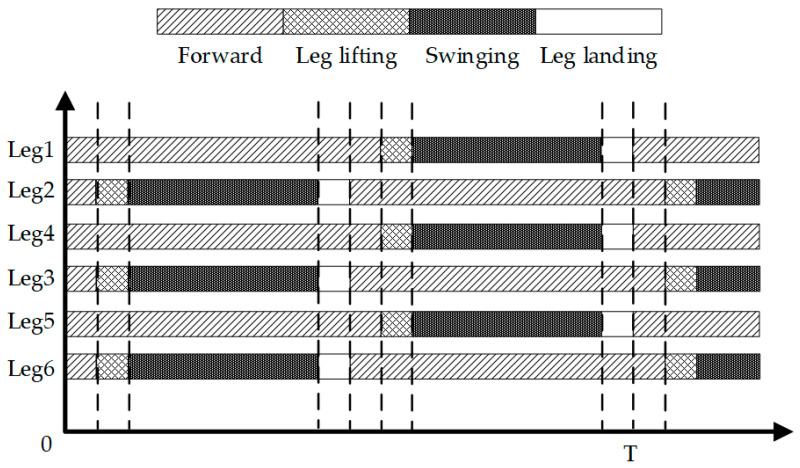
The forward gait cycle diagram of the hexapod robot.

The phases of the gait provide a clearer visualization of the alternating changes between the support and swing groups, as shown in Figure 6. The entire forward gait sequence is as follows: Initially, G1 acts as the support group and moves linearly from the starting position to the endpoint, while G2 sequentially executes the motions of leg lifting, swinging, and leg landing. Subsequently, G2 transitions to the support group, moving linearly from the starting position to the endpoint, while G1 performs the leg lifting, swinging, and leg landing. This sequence completes one fundamental cycle of the forward gait, which is then perpetually repeated to achieve continuous locomotion.

The omnidirectional gait is a specialized form of parallel displacement gait movement. Unlike the traditional forward gait movement, which follows the robot’s current orientation, the omnidirectional gait enables the hexapod robot to move in any direction without changing its current orientation. This method eliminates the need for turning and allows the hexapod robot to move more flexibly, significantly improving its maneuverability and enhancing its ability to navigate complex environments.

To achieve an omnidirectional gait movement, we maintain the robot’s current orientation while introducing the orientation deviation variable to define its movement direction, where the orientation deviation denotes the deviation between the robot’s current orientation and the target orientation. Similarly to in the forward gait, the support triangle remains stationary relative to the ground and moves in the opposite direction to the robot’s movement. The foot trajectories of the support group are parallel to the robot’s movement, with the trajectory length equal to the robot’s stride length. However, in the omnidirectional gait, the robot’s current orientation remains unchanged, while the support triangle moves in the opposite direction to both the robot’s movement and the orientation deviation. Finally, inverse kinematics is utilized to calculate the joint angles of each leg in real time. These angles are then used to control the motors driving each joint, ensuring that the feet reach their required positions, enabling the hexapod robot to achieve an omnidirectional gait.

### 4.2. Rotational Gait

**Figure 7:**
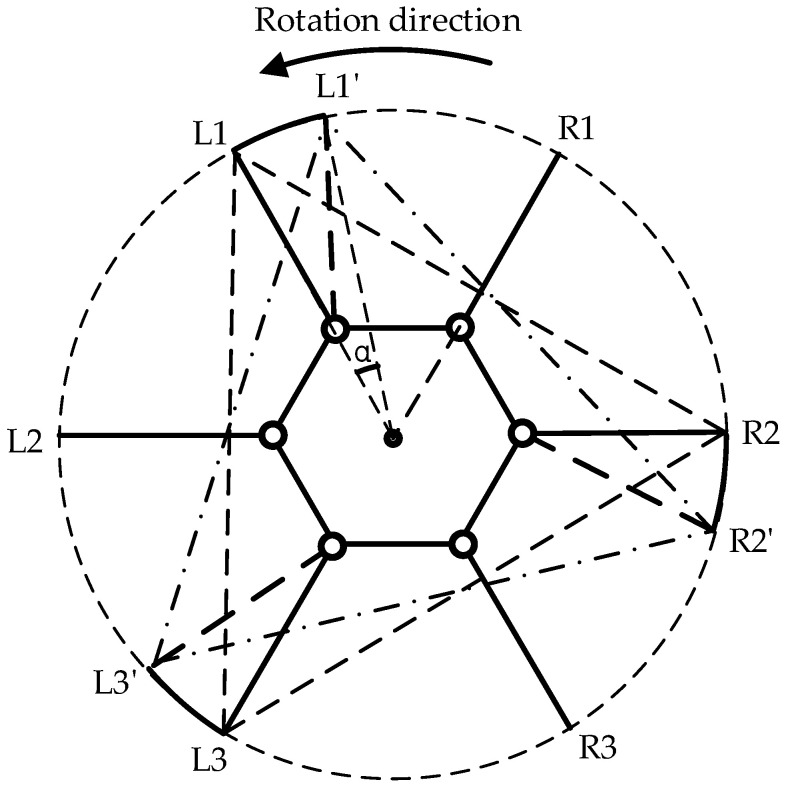
Displacement diagram of the support triangle in the rotational gait, where L1, L2, and L3 denote the endpoints of Leg1, Leg3, and Leg5, respectively, while R1, R2, and R3 represent the endpoints of Leg2, Leg4, and Leg6, respectively, and α denotes the rotation angle of the hexapod robot. The support triangle for the hexapod robot before rotational motion is formed by L1, R2, and L3, while L1′, R2′, and L3′ represent the support triangle after the robot undergoes relative movement.

The rotational gait is also a fundamental gait of the hexapod robot. Although the robot can achieve rotation by uniformly controlling all its hip joints to rotate in the same direction, we followed the forward gait method to accomplish a more precise rotational gait and complex movements. As illustrated in Figure 7, the support triangle formed by the feet of the support group serves as the focal point of analysis. In terms of relative motion, this support triangle remains stationary relative to the ground while the robot executes a rotation around its center. Consequently, the support triangle rotates around the robot’s center in the opposite direction to the robot’s rotation, maintaining an equal rotational angle to the robot’s movement. This method ensures enhanced stability and precision during the rotational gait.

**Figure 8:**
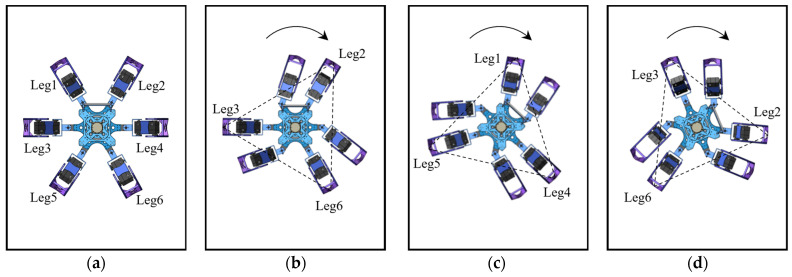
The rotational gait of the hexapod robot: (**a**) the robot’s initial state; (**b**) the robot’s status with G2 as the support group; (**c**) the robot’s status with G1 as the support group; (**d**) the robot’s status with G1 as the support group.

As shown in Figure 8, the phase of the rotational gait is similar to that of the forward gait, both completed through alternating leg support cycles. The overall rotation process begins with G1 as the support group, rotating around the robot’s center along an arc trajectory to a specified angle. During this phase, G2 sequentially executes the motions of leg lifting, swinging, and leg landing. Subsequently, G2 transitions to the support group, completing the arc trajectory, while G1 performs the leg lifting, swinging, and leg landing, thereby completing one rotational gait cycle. Sustained rotation is accomplished by perpetually repeating this basic cycle. Adjusting the number of cycles and the step angle during each rotation can precisely control the total rotation angle and speed.

### 4.3. Compound Gait

The parallel displacement gait and rotational gait are the two fundamental gaits of a hexapod robot. However, depending only on these basic gaits is often insufficient in practical applications. To address this limitation, we integrated these gaits to form more complex and flexible movement trajectories. This compound gait significantly enhances the robot’s adaptability and capability to navigate diverse and dynamic environments.

**Figure 9:**
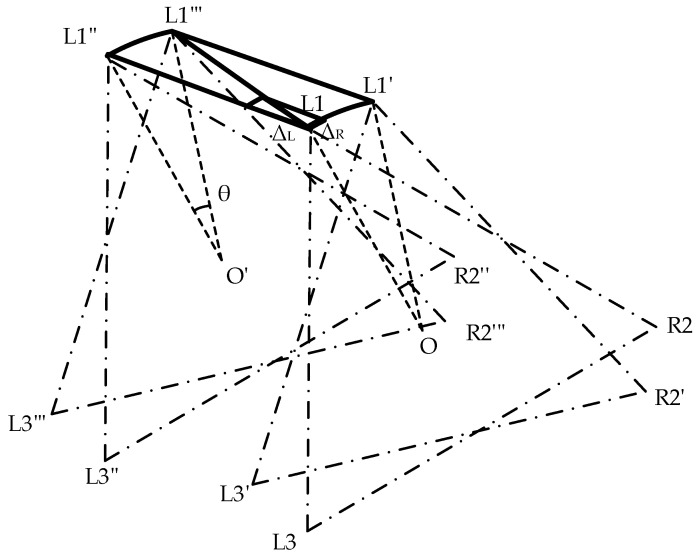
Displacement diagram of the support triangle in the compound gait, where *θ* represents the rotation angle of the hexapod robot. L1, R2, and L3 form the support triangle for the hexapod robot before compound motion, while L1‴, R2‴, and L3‴ represent the support triangle after the robot undergoes relative movement.

Assume that the hexapod robot moves from point O to point O’ while rotating its body clockwise at an angle, *θ*. In alignment with the previously detailed gait analysis, the overall movement can be effectively decomposed into a linear parallel displacement path and an in-place rotational trajectory by using the supporting triangle as the reference, as illustrated in Figure 9.

By decomposing the compound gait into independent parallel displacement and rotational paths and individually planning the trajectories for these paths, we can obtain the change vectors for each leg’s endpoint coordinates during each gait update, including the parallel displacement change vector $\Delta_{L}$ for the path L1L1″ and the rotational change vector $\Delta_{R}$ for the path L1L1′. By performing a vector addition of the parallel displacement change vector $\Delta_{L}$ and the rotational change vector $\Delta_{R}$, we can derive the incremental movement update for each step within the complex trajectory. Finally, by repeating the above steps, we can obtain the complete path curve of the feet’s endpoints under the compound gait.

### 4.4. Gait Switching Based on Fuzzy Inference

In environments with multiple obstacles and unpredictable conditions, the ability of a hexapod robot to autonomously switch gaits is essential for meeting diverse adaptive requirements and ensuring optimal performance. Whether the robot avoids obstacles or changes its direction of movement, it must adapt its gait accordingly. Most studies focus on switching from a current gait to another at the end of a current gait cycle to avoid impacts and maintain stable operation. However, in complex environments or scenarios requiring continuous directional changes, real-time switching between multiple gaits is more aligned with practical applications. The gait switching strategy model becomes significantly more complicated in these scenarios, especially in unknown environments with continuous directional changes. Li et al. [33] proposed a novel terrain-adaptive gait approach combining reinforcement learning and a CPG algorithm. This approach is treated as a partially Markov decision process (MDP). The currently obtained environment factors and robot status are fed into a pre-trained policy network with fixed parameters to calculate the trajectory of each leg in real time. However, this policy network requires a large amount of data to train better network parameters. Additionally, this method has only been tested through simulation experiments and has not been applied to real-world scenarios. However, fuzzy logic simulates the uncertain judgment and inferential thinking of the human brain. For description systems with unknown or uncertain models, a fuzzy inference controller [34,35] offers a viable solution to address these complexities.

Suppose that a hexapod robot moving forward detects an obstacle ahead. At this moment, it must use real-time gait planning to adjust its trajectory for obstacle avoidance. When all six legs are on the ground, the hexapod robot can transition to the rotational or compound gaits mentioned above or adjust the robot’s speed and movement direction. However, if the legs are alternating between support and swing phases, a sudden adjustment of the joints and foot-end trajectories following real-time gait planning may cause substantial inertial impact due to motion inertia. This direct gait switching method serves as a baseline for subsequent experimental comparisons.

For the hexapod robot’s turning or obstacle avoidance requirements, the orientation deviation can be determined by comparing the target orientation with the current orientation of the robot’s body, enabling effective maneuvering. However, in complex environments that demand continuous obstacle avoidance, seamless cornering, or sustained turning, the hexapod robot must adjust its speed dynamically. Therefore, it is also necessary to consider the velocity deviation.

For a conventional wheeled robot, the outputs of a fuzzy controller typically consist of the parallel displacement speed increments and rotational speed of the hexapod robot. However, for a hexapod robot, if the direction of the parallel displacement gait is maintained as the forward direction of the body, it will exhibit a turning radius similar to that of a wheeled robot during steering, where the turning radius refers to the radius of the smallest circle that the robot can circle when turning. Although the speed of the robot is not high in this scenario, the turning radius becomes unnecessary. By adjusting the parallel displacement gait’s direction, the hexapod robot can effectively reduce or even eliminate the turning radius, akin to a tracked vehicle’s ability to execute in-place turns. Therefore, the fuzzy inference strategy presented in this paper integrates the parallel displacement direction as a key component of the control output, thereby significantly enhancing the robot’s agility and precision in maneuvering complex environments.

**Figure 10:**
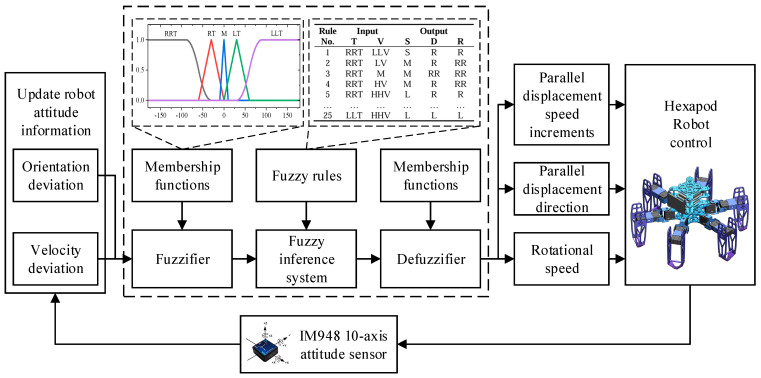
The structure of the fuzzy controller.

In summary, this study leverages orientation deviation and velocity deviation as key input variables, while output variables include the parallel displacement speed increments, parallel displacement direction, and rotational speed of the hexapod robot. This refined fuzzy inference strategy enhances the precision and agility of the robot’s movement, ensuring superior adaptability and performance in complex environments. The structure of the designed fuzzy controller is depicted in Figure 10.

The fuzzy controller’s input and output variables are fuzzified as follows. For the input variables, the orientation deviation (denoted as T) spans a range of [−180°, 180°] and is categorized into five groups: large right turn (RRT), small right turn (RT), middle (M), small left turn (LT), and large left turn (LLT). Similarly, the velocity deviation (V) is defined within the range of [−50 mm/s, 50 mm/s] and is associated with five groups: large deceleration (LLV), small deceleration (LV), middle (M), small acceleration (HV), and large acceleration (HHV). For the output variables, the parallel displacement speed increment (S) ranges from −10 mm/s to 10 mm/s and is categorized into five groups: large deceleration (LL), small deceleration (L), middle (M), small acceleration (S), and large acceleration (SS). Moreover, the parallel displacement direction (D) is similarly defined within the range of [−180°, 180°] and fuzzified into five groups: large right shift (RR), small right shift (R), middle (M), small left shift (L), and large left shift (LL). Finally, the rotational speed of the hexapod robot (R), ranging from 30°/s to 30°/s, is fuzzified into five groups: fast right turn (RR), slow right turn (R), middle (M), slow left turn (L), and fast left turn (LL).

**Figure 11:**
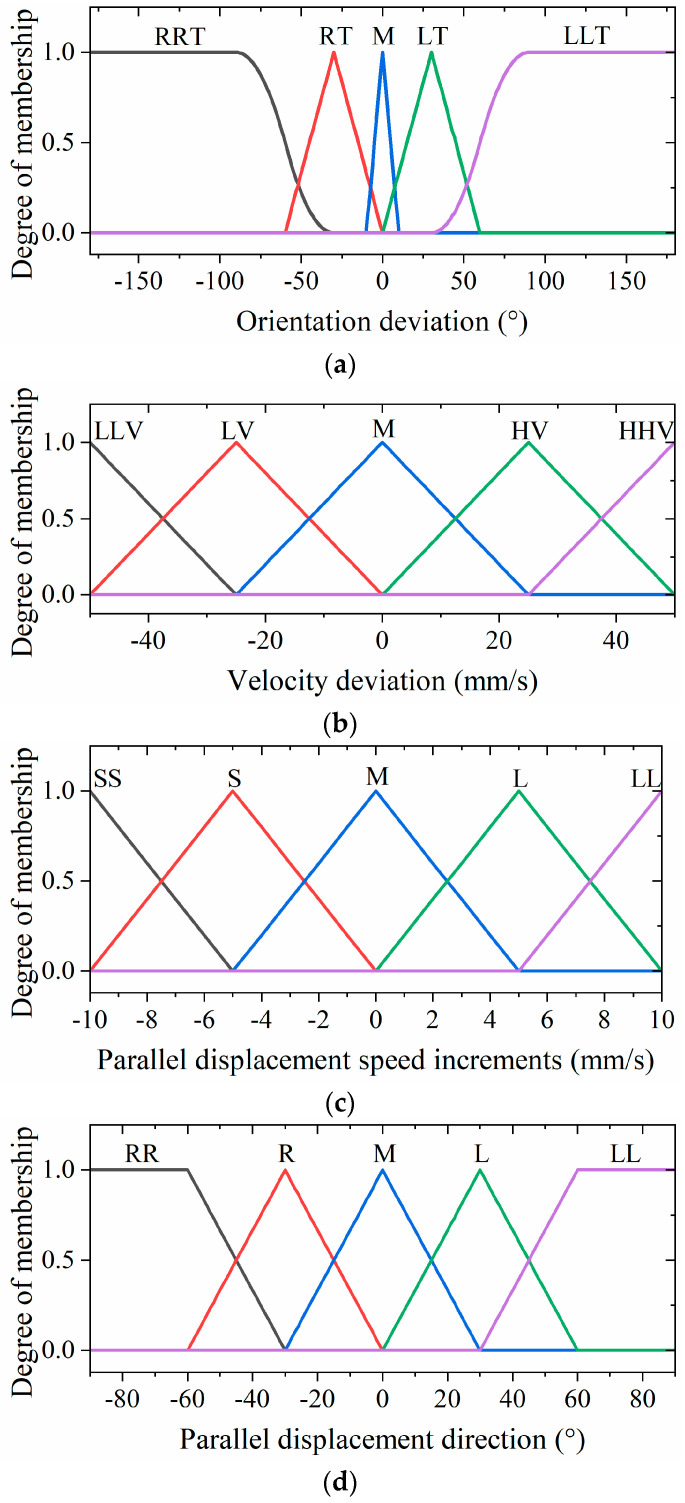

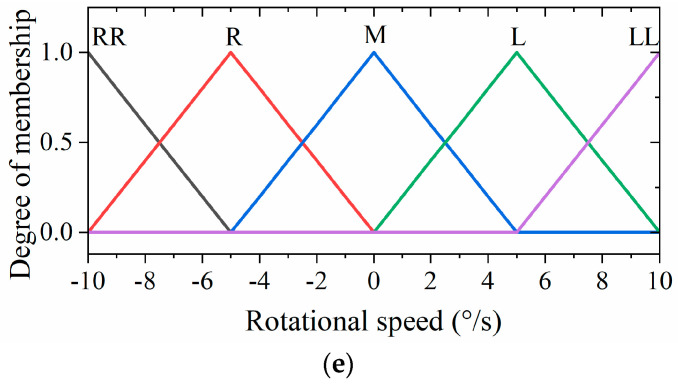
The membership functions of the (**a**) orientation deviation, (**b**) velocity deviation, (**c**) parallel displacement speed increments, (**d**) parallel displacement direction, and (**e**) rotational speed of the hexapod robot.

In order to achieve lightweight computation and smooth output, we chose S-shaped, triangular, and Z-shaped membership functions for the inputs and outputs of the fuzzy units. The membership functions of the inputs and outputs are illustrated in Figure 11.

Fuzzy inference rules constitute a critical knowledge base, directly influencing the flexibility and efficacy of the robot’s movement. These rules are crafted using expert experience and follow the conventional “IF condition, THEN result” format. Based on the number of fuzzy linguistic terms associated with the input variables, a total of 25 fuzzy inference rules can be obtained to precisely dictate the robot’s movement dynamics, which can be expressed as follows:

IF (T is Ti and V is Vi) THEN (S is Si and D is Di and R is Ri)

where Ti, Vi, Si, Di, and Ri are fuzzy inference sets defined on T, V, S, D, and R, respectively. The fuzzy inference rules are illustrated in Table 3.

**Figure 12:**
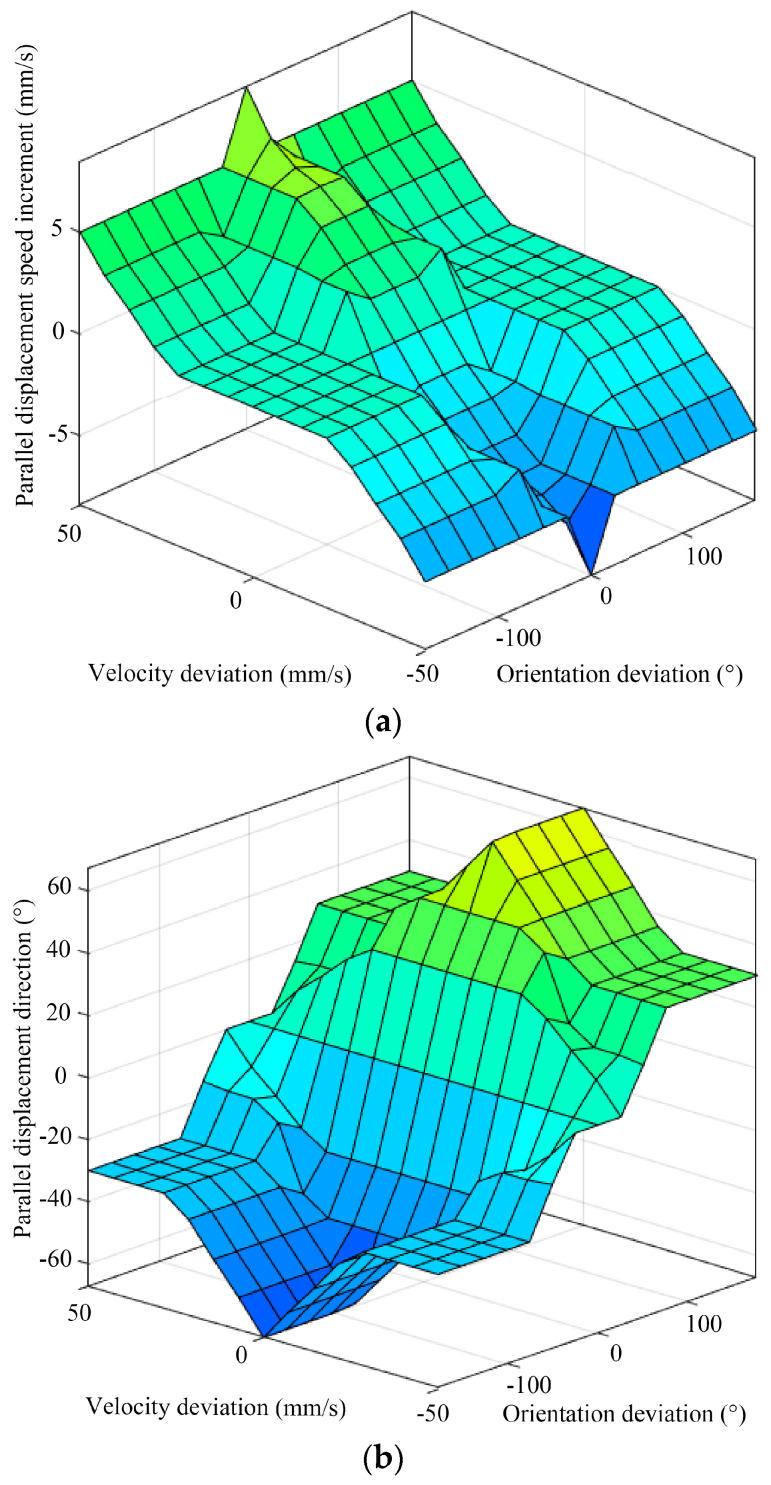

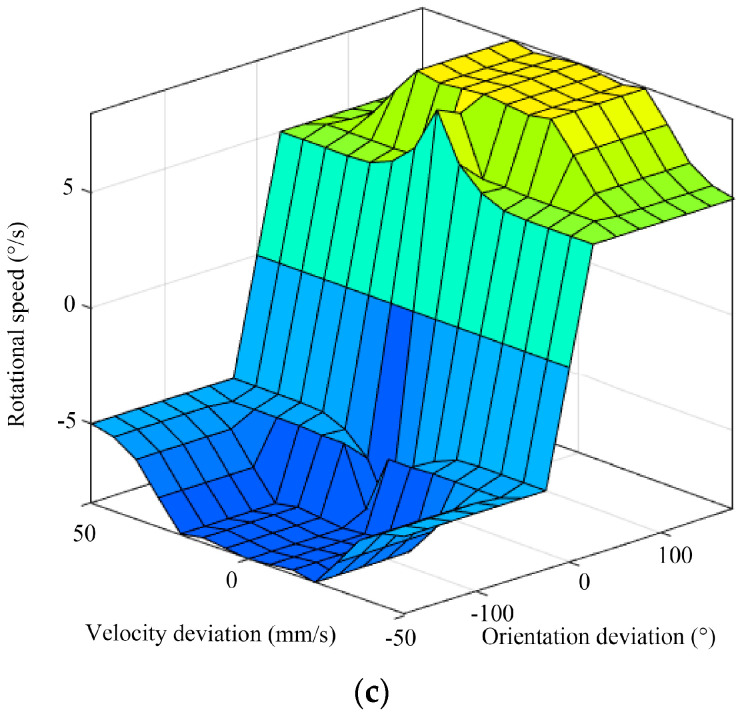
The fuzzy rule surface of the (**a**) parallel displacement speed increments, (**b**) parallel displacement direction, and (**c**) rotational speed of the hexapod robot.

Based on the established membership functions and fuzzy inference rules, the inference output of the fuzzy controller can be obtained. The center-of-gravity method is employed for defuzzification to obtain the results of the fuzzy inference. Figure 12 illustrates the fuzzy rule surface of the hexapod robot.

## 5. Attitude Control Strategy

Attitude control is crucial for hexapod robots, as it directly impacts their stability, maneuverability, and adaptability across various environments. By precisely controlling the robot’s movement direction, gait, and attitude, all six legs can maintain optimal contact with the ground, providing balanced support and stable propulsion. This is particularly important on uneven or complex terrain, where the robot’s ability to adjust its attitude in real time prevents tipping, enhances traction, and improves movement efficiency. Moreover, effective attitude control facilitates seamless transitions between different gaits, which are essential for adapting to dynamic conditions or performing complex tasks, such as search-and-rescue missions or exploration missions in dangerous situations. In specialized applications, attitude control can be integrated with sensory inputs and adaptive algorithms, enabling the robot to autonomously adjust its attitude in response to environmental changes, thereby enhancing its operational robustness and versatility.

### 5.1. Attitude Control Mapping Model

**Figure 13:**
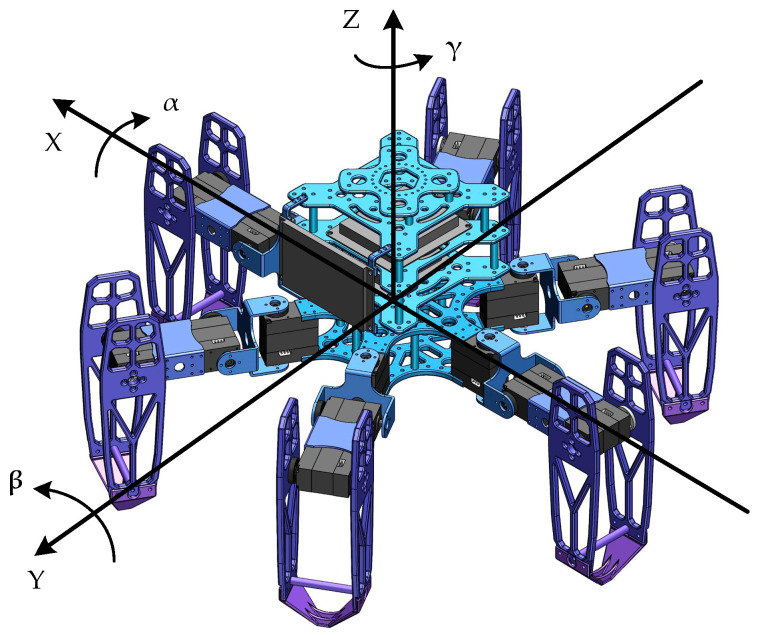
The attitude angles diagram for the hexapod robot.

During the walking process, the hexapod robot plans its movement trajectory based on predefined walking parameters and then generates the corresponding leg trajectories. Meanwhile, the robot controls its attitude during movement by adjusting the joint angles of its supporting legs. The range of adjustable attitude for the robot is determined by its structural parameters and joint angles. The fixed position of the supporting legs’ foot-ends influences the range of joint angle variations. Moreover, the foot-ends’ coordinates are defined with the chassis center as the reference origin and the chassis plane as the reference plane. When the global coordinates of the foot-end remain constant, any adjustment in the robot’s attitude alters the foot-end coordinates within the robot’s reference frame. Consequently, the attitude adjustments of the robot are reflected as changes in the foot-end coordinates. By establishing a dynamic mapping relationship between the foot-end positions and the robot’s attitude, the robot can adjust from its current attitude to the target attitude at any given moment. Figure 13 illustrates the attitude angles diagram for the hexapod robot.

The hexapod robot uses a gyroscope to measure its actual attitude angles, including the pitch angle around the x-axis, the roll angle around the y-axis, and the yaw angle around the z-axis. This study discusses the robot’s horizontal posture without considering the yaw angle, allowing us to conserve computational resources during attitude adjustments. Thus, the robot’s attitude angles can be decomposed into the pitch angle α around the x-axis and the roll angle β around the y-axis. Therefore, the hexapod robot can achieve its pitch and roll angle adjustments by modifying the coordinates of the joint endpoints relative to the center of the chassis. Then, we can obtain the general attitude adjustment matrix, expressed as follows:

(8) $$\begin{array}{ccc} R\left(\alpha ,\beta \right) & =R_{y}\left(\beta \right)R_{x}\left(\alpha \right)\\ & =\left[\begin{array}{ccc} \cos\beta & 0 & \sin\beta \\ 0 & 1 & 0\\ -\sin\beta & 0 & \cos\beta \end{array}\right]\left[\begin{array}{ccc} 1 & 0 & 0\\ 0 & \cos\alpha & -\sin\alpha \\ 0 & \sin\alpha & \cos\alpha \end{array}\right] & \\ & =\left[\begin{array}{ccc} \cos\beta & \sin\beta \sin\alpha & \sin\beta \cos\alpha \\ 0 & \cos\alpha & -\sin\alpha \\ -\sin\beta & \cos\beta \sin\alpha & \cos\beta \cos\alpha \end{array}\right] \end{array}$$

Suppose that $P_{0}\left(x_{0},y_{0},z_{0}\right)$ denotes the foot-end coordinates before attitude adjustment, and $P\left(x,y,z\right)$ represents the new coordinates after attitude adjustment. Thus, we can obtain the new coordinates $P\left(x,y,z\right)$ after attitude adjustment as follows:

(9) $$\begin{array}{cc} \left[\begin{array}{c} x\\ y\\ z \end{array}\right] & =R_{y}\left(\beta \right)R_{x}\left(\alpha \right)\left[\begin{array}{c} x_{0}\\ y_{0}\\ z_{0} \end{array}\right]\\ & =\left[\begin{array}{c} x_{0}\cos\beta +y_{0}\sin\beta \sin\alpha +z_{0}\sin\beta \cos\alpha \\ y_{0}\cos\alpha -z_{0}\sin\alpha \\ -x_{0}\sin\beta +y_{0}\cos\beta \sin\alpha +z_{0}\cos\beta \cos\alpha \end{array}\right] \end{array}$$

Attitude adjustment can be regarded as the active adjustment of the robot, which is not affected by the ground. In order to ensure smooth and stable attitude adjustments, the transition from the initial attitude to the target attitude is achieved by using an interpolation method to plan the attitude angle change curve.

### 5.2. Attitude Control Strategy Based on Single-Neuron Adaptive PID

The proportional–integral–derivative (PID) technique is a widely used feedback control mechanism in automatic control systems due to its simplicity and effectiveness [36,37]; it is applied across various industries, including robotics, process control, and motor systems. The incremental PID [38] is a variant of the conventional PID controller and can be applied for real-time attitude adjustments in robots; its mathematical expression is as follows:

(10) $$\Delta u_{k}=K_{p}\cdot \left(e_{k}-e_{k-1}\right)+K_{i}\cdot e_{k}+K_{d}\cdot \left(e_{k}-2e_{k-1}+e_{k-2}\right)$$

where $\Delta u_{k}$ denotes the control output increment at the current moment; $K_{p}$, $K_{i}$, and $K_{d}$ are the proportional, integral, and derivative coefficients of the PID parameters, respectively; and $e_{k}$ is the input error between the desired output and the actual output in the *k*-th sampling.

However, the proper tuning of the PID parameters can be challenging, especially for complex systems with multiple interacting variables. Additionally, it cannot adapt to changes in the system, particularly under conditions of significant disturbances or parameter variations. Therefore, the PID algorithm is not well suited for direct application in the attitude control systems of hexapod robots, especially when operating in complex and dynamic external environments.

**Figure 14:**
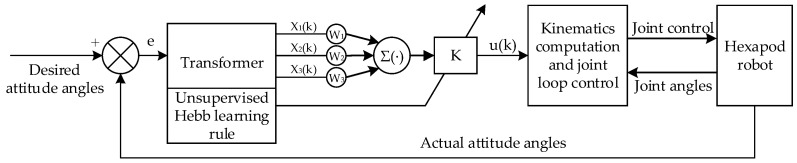
The architecture of the attitude control strategy.

The single-neuron adaptive PID control algorithm combines the traditional PID controller with a single-neuron model, leveraging the neuron’s self-learning and adaptive capabilities [39,40]. The neuron processes error signals and their derivatives, iteratively learning to minimize errors, thereby enabling the real-time intelligent adjustment of the PID parameters to enhance adaptive capabilities. Compared to traditional PID control, this method can more effectively handle complex dynamic systems with continuously varying parameters or disturbances. Therefore, an attitude control strategy based on the single-neuron adaptive PID algorithm is proposed for the hexapod robot in this paper. Figure 14 illustrates the structure of the attitude control strategy.

For the adjustment of the horizontal attitude of the hexapod robot, the desired and actual attitude angles are used as input signals for the controller. After transformation, these signals yield the state variables x_1_, x_2_, and x_3_, which are required by the single-neuron adaptive controller. This attitude control strategy utilizes the incremental PID algorithm, and the state variables can be expressed as follows:

(11) $$\left\{\begin{array}{l} x_{1}\left(k\right)=e_{k}-e_{k-1}\\ x_{2}\left(k\right)=e_{k}\\ x_{3}\left(k\right)=e_{k}-2e_{k-1}+e_{k-2} \end{array}\right.$$

The proportional, integral, and derivative coefficients in the PID parameters are treated as corresponding weight coefficients, denoted by $w_{i}\left(k\right)$. Additionally, a proportional coefficient K is introduced. Therefore, the formula for the single-neuron controller based on the incremental PID algorithm is as follows:

(12) $$u_{k}=u_{k-1}+K\sum_{i=1}^{3}{w_{i}}^{\prime }\left(k\right)x_{i}\left(k\right)$$

Learning is a fundamental characteristic of neural networks, and learning rules are the primary mechanism for achieving this process. These rules primarily adjust the connection weights between neurons, effectively modifying the weights. In this paper, the unsupervised Hebb learning rule [41] is adopted, yielding the single-neuron adaptive PID algorithm as follows:

(13) $$\left\{\begin{array}{l} u_{k}=u_{k-1}+K\sum_{i=1}^{3}{w_{i}}^{\prime }\left(k\right)x_{i}\left(k\right)\\ {w_{i}}^{\prime }\left(k\right)=w_{i}\left(k\right)/\sum_{i=1}^{3}\left|w_{i}\left(k\right)\right|\\ w_{1}\left(k\right)=w_{1}\left(k-1\right)+\eta_{P}z\left(k\right)u\left(k\right)x_{1}\left(k\right)\\ w_{2}\left(k\right)=w_{2}\left(k-1\right)+\eta_{I}z\left(k\right)u\left(k\right)x_{2}\left(k\right)\\ w_{3}\left(k\right)=w_{3}\left(k-1\right)+\eta_{D}z\left(k\right)u\left(k\right)x_{3}\left(k\right)\\ z\left(k\right)=e_{k} \end{array}\right.$$

where $\eta_{P}$, $\eta_{I}$, and $\eta_{D}$ are the learning rates of proportion, integration, and differentiation, respectively. By applying the backpropagation formula through the network, the weight coefficients are updated. After normalization, the adaptive parameters $K_{P}$, $K_{I}$, and $K_{D}$ for the single-neuron adaptive PID can be obtained.

## 6. Experiments and Results

In order to verify the effectiveness of our proposed algorithm, three sets of test experiments were conducted. These experiments encompassed various gait motion experiments, gait switching motion experiments, and attitude control experiments.

### 6.1. Gait Motion Experiments

**Figure 15:**
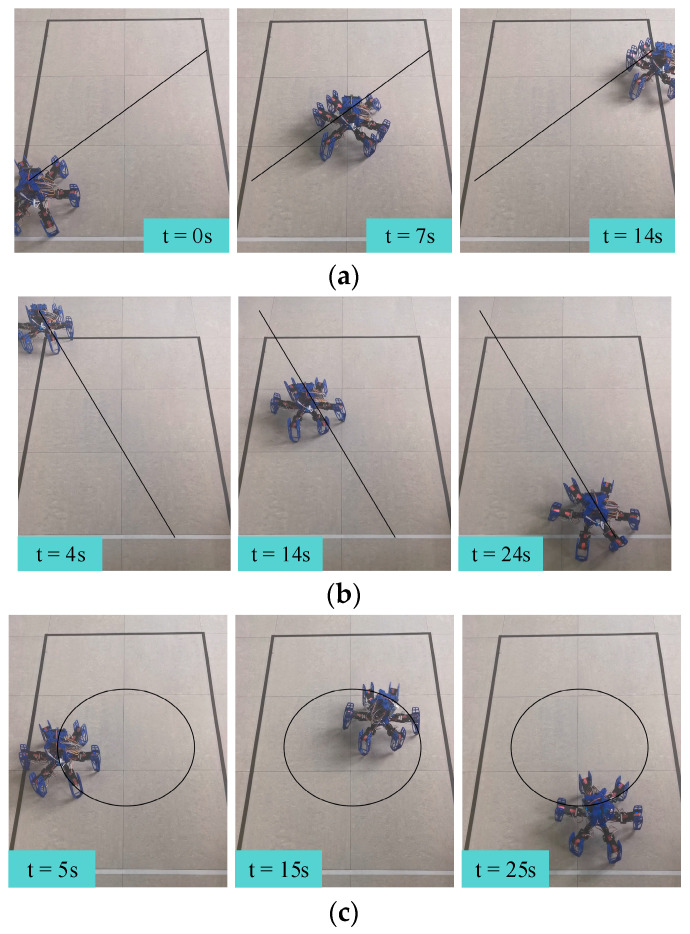
Snapshots of each gait test, with time labels: (**a**) omnidirectional gait with 45° orientation deviation, (**b**) omnidirectional gait with 150° orientation deviation, and (**c**) rotational gait.

In order to validate the performance of the various proposed gaits, we conducted three sets of gait motion experiments. First, two omnidirectional movement experiments were carried out, maintaining the hexapod robot’s current orientation while setting the orientation deviation to 45° and 150°, respectively. The snapshots of these two gait experiments with time labels are depicted in Figure 15a,b. Finally, we performed the third experiment, where the hexapod robot executed a circular motion with a defined radius, as shown in Figure 15c.

Figure 15a,b demonstrate that the hexapod robot could execute omnidirectional movement according to the predefined target orientation deviation while maintaining its current orientation. Furthermore, by analyzing the hexapod robot’s position relative to the reference line, we can see that the distance between the hexapod robot and the reference line remains nearly constant, confirming that the hexapod robot followed a straight path in the direction of the predefined orientation deviation, further validating the effectiveness of the omnidirectional gait. As shown in Figure 15c, the hexapod robot could continuously adjust its orientation and perform circular motion with a specified radius using the proposed compound gait. This successfully demonstrated the effectiveness of the compound gait.

### 6.2. Gait Switching Experiments

In order to assess the performance of our proposed gait switching algorithm, we conducted three sets of experiments using different gait switching strategies. These strategies included direct gait switching, a fuzzy-inference-based gait switching strategy, and a combined omnidirectional and fuzzy-inference-based gait switching strategy. In the second set of experiments, the fuzzy-inference-based strategy did not include omnidirectional gaits, so orientation deviation was not considered. To evaluate the stability of the hexapod robot, we employed attitude angles that directly impact stability, including the pitch and roll angles. These angles were introduced as primary metrics for assessing the robot’s stability [23]. *Stability* is defined as the robot’s equilibrium, while *D* (*stability*) represents the variance in *stability*, which can be expressed as follows [23]:

(14) $$stability=\frac{m\left|\alpha \right|+n\left|\beta \right|}{t}$$

(15) $$D(stability)=D(\frac{m\left|\alpha \right|+n\left|\beta \right|}{t})$$

where α and β represent the pitch and roll angles of the hexapod robot, respectively; *m* and *n* denote the weight of the pitch and roll angles, respectively; and *t* is the sample time. Considering that the roll and pitch angles are equally important in this paper, we set *m* and *n* to 1.0. Therefore, the smaller the value of *D* (*stability*), the better the stability of the hexapod robot.

**Figure 16:**
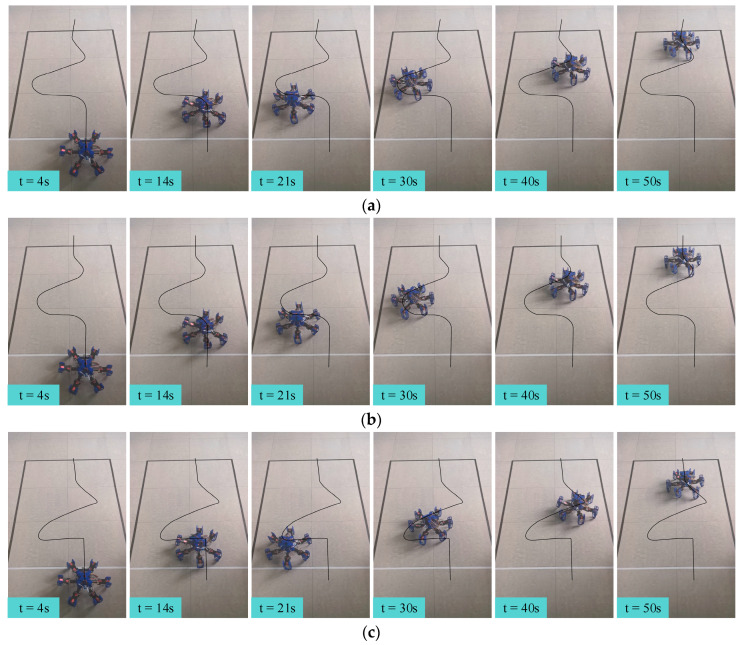
The snapshots of each gait switching algorithm with time labels: (**a**) the direct gait switching strategy, (**b**) the fuzzy-inference-based gait switching strategy, and (**c**) the combined omnidirectional and fuzzy-inference-based gait switching strategy.

**Figure 17:**
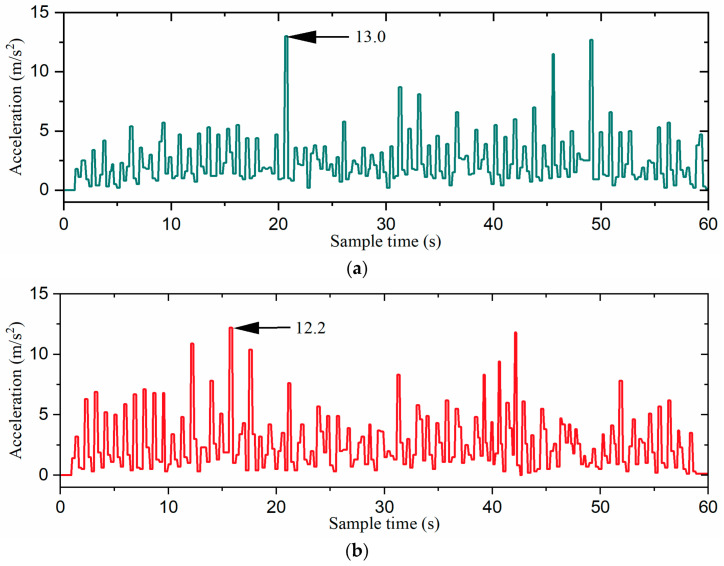

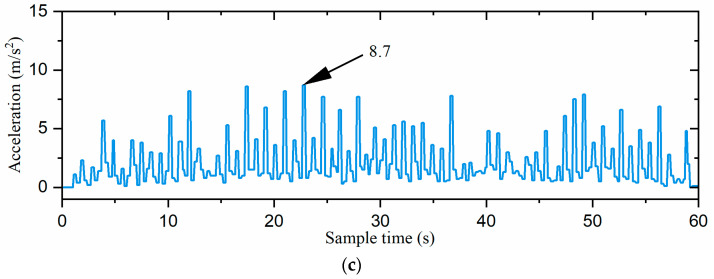
The acceleration curves of the robot: (**a**) the direct gait switching strategy, (**b**) the fuzzy-inference-based gait switching strategy, and (**c**) the combined omnidirectional and fuzzy-inference-based gait switching strategy. The acceleration is the absolute value of the inertial acceleration.

**Figure 18:**
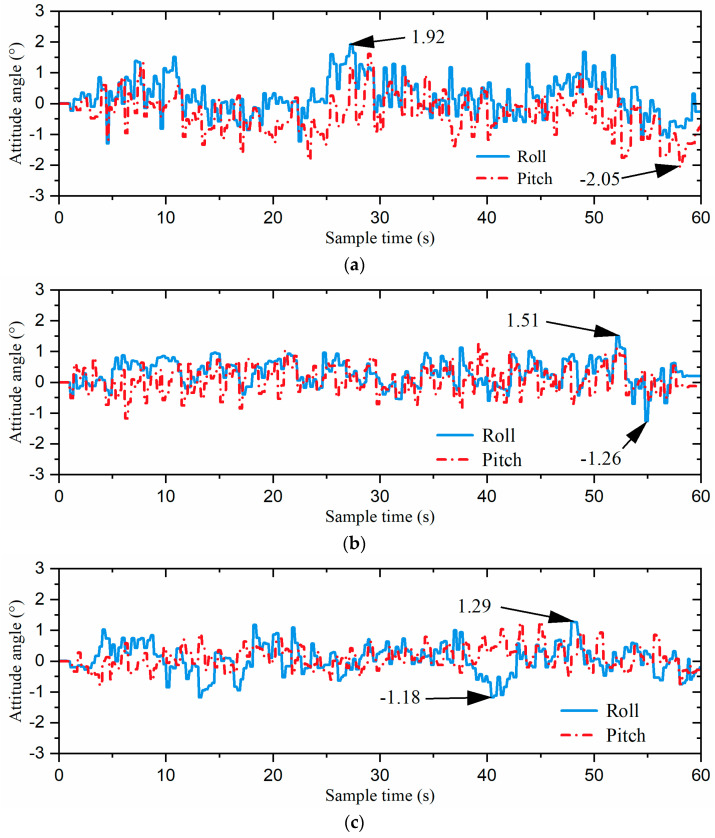
The attitude curves of the robot: (**a**) the direct gait switching strategy, (**b**) the fuzzy-inference-based gait switching strategy, and (**c**) the combined omnidirectional and fuzzy-inference-based gait switching strategy.

The gait switching task involved moving from the starting point at a speed of 5 cm/s in the 0° direction of the global coordinate system for 9 s (task1), followed by 9 s of movement at the same speed in the −90° direction (task2). Then, the robot moved in the 60° direction for 16.2 s at 5 cm/s (task3), followed by 9 s in the −30° direction (task4), and finally another 9 s in the 0° direction (task5), all including gait switching times. The snapshots of each gait switching algorithm with time labels are shown in Figure 16. Additionally, the acceleration curves and attitude curves for the three gait switching experiments are shown in Figure 17 and Figure 18, respectively. It is worth mentioning that the acceleration and attitude angles obtained by the sensor were processed by noise filtering.

Figure 16a,b show that the turning radius using the direct gait switching strategy was slightly smaller than that of the fuzzy-inference-based gait switching strategy. This is because the required turning angle was evenly distributed across the designated cycles in the direct switching strategy. In contrast, the fuzzy-inference-based gait switching strategy randomly distributed the turning angle across cycles through fuzzy inference, requiring more cycles and resulting in a slightly larger turning radius. When the omnidirectional gait was introduced, the turning radius of the combined omnidirectional and fuzzy-inference-based gait switching strategy was significantly smaller than that of the previous two strategies, as illustrated in Figure 16c. This improvement was primarily due to the hexapod robot’s ability to move directly toward the target direction using the omnidirectional gait, avoiding the need for compound movements along its current orientation, thereby reducing the turning radius.

In order to further verify the experimental results mentioned above, we extracted the robot’s trajectory path and applied numerical fitting to the turning radius during certain gait transitions. This allowed us to calculate the minimum turning radius of each gait switching strategy in the gait switching process, as shown in Table 4. As can be seen from the table, the combined omnidirectional and fuzzy-inference-based gait switching strategy had the smallest turning radius in each gait switch, which further verifies the above experimental conclusions. Therefore, our proposed combined omnidirectional and fuzzy-inference-based gait switching strategy provided the hexapod robot with more flexible and accurate gait control, significantly enhancing its maneuverability.

As shown in Figure 17 and Figure 18, the direct gait switching strategy resulted in high acceleration and large attitude angle fluctuations, causing a significant impact on the hexapod robot. However, when using the fuzzy-inference-based gait switching algorithm, the hexapod robot experienced reduced acceleration and less fluctuation in the attitude angles. With the combined omnidirectional and fuzzy-inference-based gait switching strategy, the hexapod robot’s acceleration was significantly reduced, and its attitude angle fluctuation was smaller than under the previous two strategies. In addition, compared to the previous two strategies, our proposed strategy reduced the hexapod robot’s acceleration by 33.1% and 28.7% and the maximum attitude angle by 32.8% and 14.6%, respectively. That is, the hexapod robot experienced smoother acceleration, which reduced the inertial impact on the body and improved the overall stability of the hexapod robot.

**Figure 19:**
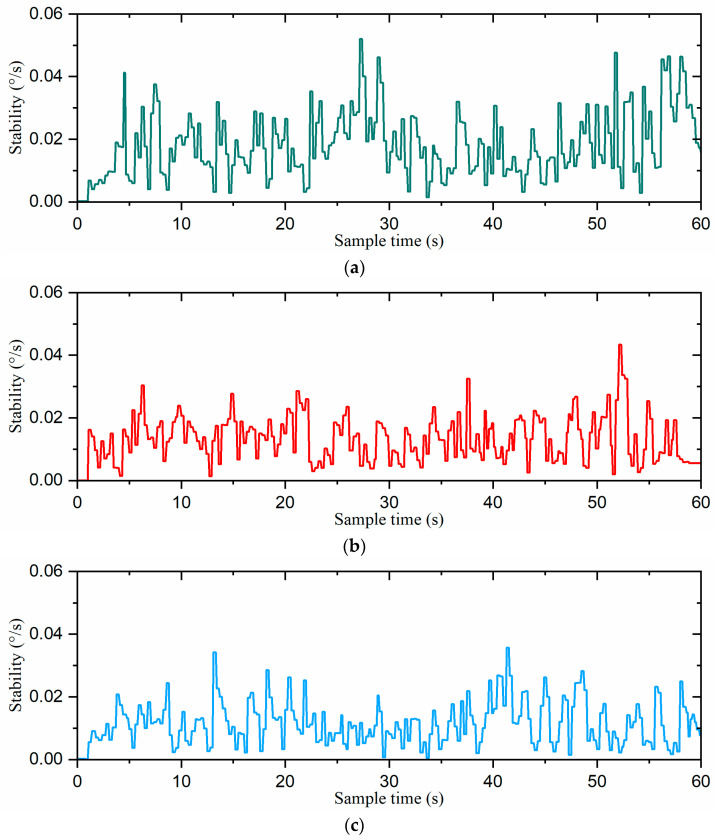
The stability curves of the robot: (**a**) the direct gait switching strategy, (**b**) the fuzzy-inference-based gait switching strategy, and (**c**) the combined omnidirectional and fuzzy-inference-based gait switching strategy.

The stability curves of the robot for the three gait switching strategies are shown in Figure 19. The *stability* of the direct gait switching strategy ranged from 0°/s to 0.052°/s. With the fuzzy-inference-based gait switching strategy, the *stability* range was relatively small, fluctuating between 0°/s and 0.043°/s. However, under the combined omnidirectional and fuzzy-inference-based gait switching strategy, the *stability* range was further compressed to 0°/s to 0.036°/s. These results demonstrate that our combined omnidirectional and fuzzy-inference-based strategy enhanced the robot’s stability during gait switching.

To further validate the performance of the hexapod robot’s stability, we calculated D (stability) using Equations (14) and (15), with the results summarized in Table 5. With the fuzzy-inference-based gait switching strategy, *D (stability)* was 10.72 × 10^−3^. When the fuzzy-inference-based gait switching strategy was employed, *D (stability)* was reduced to 7.40 × 10^−3^. However, under the combined omnidirectional and fuzzy-inference-based gait switching strategy, *D (stability)* was further improved to 7.03 × 10^−3^. Compared to the previous two strategies, our proposed approach improved the D (stability) value of the hexapod robot by 34.4% and 5.0%, respectively. Therefore, our proposed combined omnidirectional and fuzzy-inference-based gait switching strategy improved the stability of the hexapod robot during gait switching.

### 6.3. Attitude Control Experiments

Attitude control experiments were carried out to assess the effectiveness of our proposed attitude control algorithm based on the single-neuron adaptive PID. We adopted the incremental PID attitude control method as a baseline and conducted two sets of attitude adjustment experiments. In the experiments, the hexapod robot was first initialized on a flat table surface to set its attitude. Then, it was placed on a slope, with its body parallel to the incline. After 2 s, both attitude control strategies were automatically activated to quickly adjust the hexapod robot’s attitude, ensuring that its body returned to a level position parallel to the table surface. It is worth mentioning that the attitude angles obtained by the sensor were processed by noise filtering.

**Figure 20:**
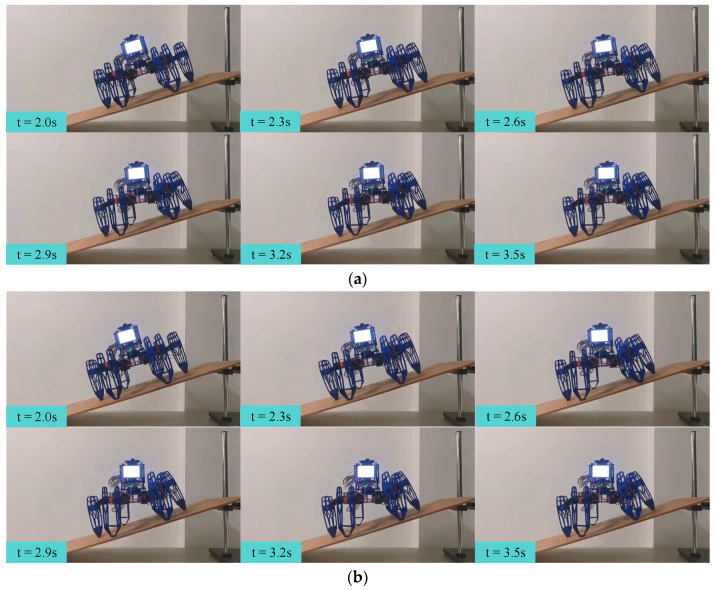
Snapshots of each attitude control algorithm, with time labels: (**a**) incremental PID and (**b**) single-neuron adaptive PID.

Figure 20 provides snapshots of the two attitude control algorithms, with time labels. The figure shows that both algorithms enabled the hexapod robot to adjust its attitude angles quickly, within 1.5 s.

**Figure 21:**
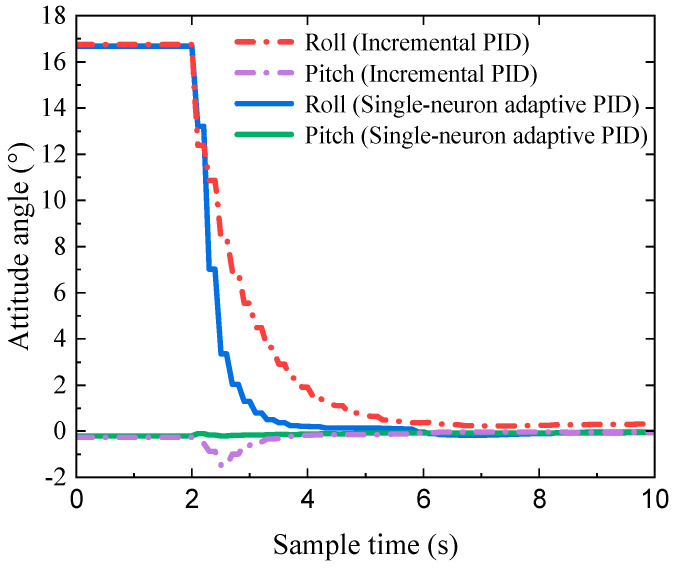
The attitude control curves of the incremental PID and single-neuron adaptive PID.

Figure 21 presents the attitude control curves during the experiments. It is clearly shown that the incremental PID control strategy took approximately 4 s to reach the desired attitude. In comparison, the single-neuron adaptive PID attitude control strategy achieved this in less than 2 s, reducing the attitude adjustment time by 50%. The experimental data reveal that the adaptive single-neuron attitude control strategy achieved faster adjustments to the predefined attitude, demonstrating superior performance in attitude control.

### 6.4. Slope-Climbing Experiments

To demonstrate the performance of dynamic attitude control, we conducted slope-climbing experiments with the body parallel to the horizontal plane. We adopted the incremental PID attitude control method as a baseline and conducted two sets of slope-climbing experiments. In the experiments, the hexapod robot was initialized on a horizontal plane to set its attitude. Then, it was placed at the bottom of the slope. After the experiment started, the hexapod robot was controlled to walk on the slope toward the top of the slope, while keeping its body parallel to the horizontal plane during the walking process. It is worth mentioning that the attitude angles obtained by the sensor were processed by noise filtering.

**Figure 22:**
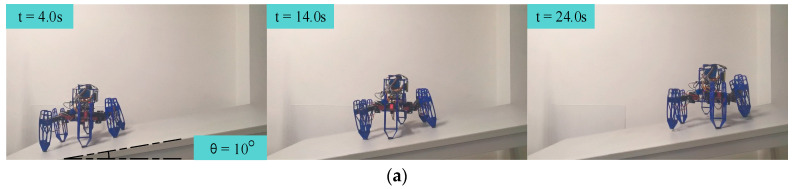

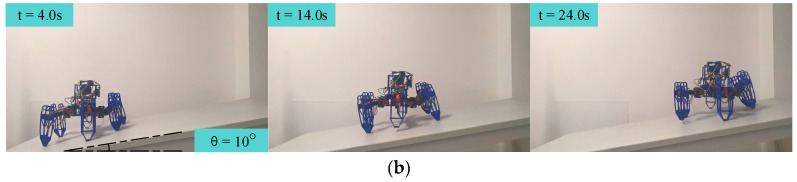
Snapshots of each attitude control algorithm for the slope-climbing experiments, with the body parallel to the horizontal plane: the (**a**) incremental PID and (**b**) single-neuron adaptive PID.

Snapshots of each attitude control algorithm for the slope-climbing experiments, with the body parallel to the horizontal plane, are shown in Figure 22. As can be seen from the figure, both strategies could enable the hexapod robot to keep its body parallel to the horizontal plane and climb from the bottom to the top of the slope.

**Figure 23:**
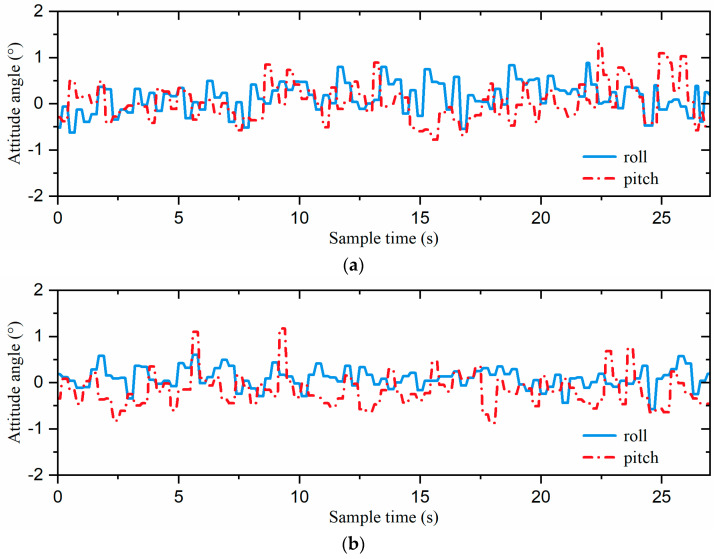
The attitude curves of the robot: the (**a**) incremental PID and (**b**) single-neuron adaptive PID.

The attitude curves of each attitude control algorithm for the slope-climbing experiments, with the body parallel to the horizontal plane, are shown in Figure 23. The attitude angles of the incremental PID strategy ranged from −0.78° to 1.30°. With the single-neuron adaptive PID strategy, the range of attitude angles was relatively small, fluctuating between −0.87° and 1.17°.

In order to further validate the performance of the hexapod robot’s stability, we calculated D (stability) using Equations (14) and (15), with the results summarized in Table 6. With the incremental PID strategy, *D* (*stability*) was 11.41 × 10^−3^. However, when the single-neuron adaptive PID strategy was employed, *D* (*stability*) was reduced to 10.74 × 10^−3^. Our approach achieved a 5.9% enhancement in *D* (*stability*).

Although the slope-climbing experiments were conducted on a slope with an angle of 10°, they demonstrated that the hexapod robot could climb the slope and dynamically adjust its attitude in real time. In addition, experimental data also show that the adaptive single-neuron attitude control strategy could better maintain attitude stability during climbing and exhibited superior performance in dynamic attitude control.

## 7. Conclusions

This work proposed a gait switching approach based on a combined omnidirectional and fuzzy inference model and a single-neuron adaptive PID attitude control method for a hexapod robot. First, we took advantage of the hexapod robot’s kinematic model to introduce an omnidirectional gait, aimed at addressing the challenge of precise trajectory control under limited stride and steering conditions. Next, to tackle the issue of poor stability during real-time random gait switching, we presented a real-time replanning gait switching strategy based on omnidirectional gait and fuzzy inference. Finally, to further enhance the stability of the hexapod robot, an attitude adjustment algorithm based on the single-neuron adaptive PID was proposed. A series of experiments were conducted to validate the effectiveness of the proposed approach. During the gait switching experiments, the hexapod robot was able to change direction rapidly and achieved precise trajectory control using the omnidirectional gait switching strategy based on fuzzy inference. Additionally, in attitude adjustment experiments conducted on an 18° slope, the single-neuron adaptive PID attitude control algorithm enabled the hexapod robot to reach the desired attitude quickly.

In future work, we plan to equip the foot-ends with force feedback sensors to enhance the hexapod robot’s ability to adapt to various terrain through foot–ground interactions, thereby improving its motion stability and environmental adaptability. Furthermore, we intend to integrate external environmental sensing capabilities to enable the hexapod robot to make autonomous decisions.

## Figures and Tables

**Table 1:** The kinematic parameters of the proposed hexapod robot.

Name	Attributes
Size	480 mm (L) × 480 mm (W) × 310 mm (H)
Weights	2.7 kg
Degrees of freedom	18
The length of the base joint (L_1_)	48 mm
The length of the thigh (L_2_)	72 mm
The length of the shin (L_3_)	144 mm
The rotation range of the hip joint	[−90°, 90°]
The rotation range of the knee joint	[−90°, 90°]
The rotation range of the ankle joint	[−135°, 135°]

**Table 2:** The D-H parameters of one leg of the proposed hexapod robot.

i	$\theta_{i}$ (°)	$d_{i}$ (mm)	$a_{i}$ (mm)	$\alpha_{i}$ (°)
1	$\theta_{1}$	0	$L_{1}$	90
2	$\theta_{2}$	0	$L_{2}$	0
3	$\theta_{3}$	0	$L_{3}$	0

Here, $\theta_{i}$ denotes the angle, which rotates around the Z_i−1_ axis to align X_i−1_ with the X_i_ direction; $d_{i}$ represents the offset distance along the Z_i−1_ axis to ensure that X_i−1_ remains collinear with X_i_; $a_{i}$ specifies the link length, translating along the X_i_ axis from Z_i−1_ to Z_i_ axes; and $\alpha_{i}$ represents the rotation angle around X_i_, aligning the Z_i−1_ and Z_i_ axes.

**Table 3:** The proposed fuzzy inference rules.

Rule No.	Input		Output		
	T	V	S	D	R
1	RRT	LLV	S	R	R
2	RRT	LV	M	R	RR
3	RRT	M	M	RR	RR
4	RRT	HV	M	R	RR
5	RRT	HHV	L	R	R
6	RT	LLV	S	M	R
7	RT	LV	S	R	R
8	RT	M	M	R	RR
9	RT	HV	L	R	R
10	RT	HHV	L	M	R
11	M	LLV	SS	M	M
12	M	LV	S	M	M
13	M	M	M	M	M
14	M	HV	L	M	M
15	M	HHV	LL	M	M
16	LT	LLV	S	M	L
17	LT	LV	S	L	L
18	LT	M	M	L	LL
19	LT	HV	L	L	L
20	LT	HHV	L	M	L
21	LLT	LLV	S	L	L
22	LLT	LV	M	L	LL
23	LLT	M	M	LL	LL
24	LLT	HV	M	L	LL
25	LLT	HHV	L	L	L

**Table 4:** The minimum turning radius during each gait switching task.

Gait Switching Task	Direct Gait Switching	Fuzzy-Inference-Based Gait Switching	Combined Omnidirectional and Fuzzy-Inference-Based Gait Switching
Task1 to task2	303 mm	369 mm	14 mm
Task2 to task3	272 mm	373 mm	117 mm
Task3 to task4	178 mm	353 mm	39 mm

**Table 5:** The variance in stability for the three gait switching strategies.

Evaluation Metrics	Direct Gait Switching	Fuzzy-Inference-Based Gait Switching	Combined Omnidirectional and Fuzzy-Inference-Based Gait Switching
D (stability) (×10^−3^)	10.72	7.40	7.03

**Table 6:** The variance in stability for each attitude control algorithm.

Evaluation Metrics	Incremental PID	Single-Neuron Adaptive PID
D (stability) (×10^−3^)	11.41	10.74

## Data Availability

The data presented in this study are available upon request from the corresponding author.
